# Whole Genome Analysis of Two Novel Type 2 Porcine Reproductive and Respiratory Syndrome Viruses with Complex Genome Recombination between Lineage 8, 3, and 1 Strains Identified in Southwestern China

**DOI:** 10.3390/v10060328

**Published:** 2018-06-15

**Authors:** Long Zhou, Runmin Kang, Yi Zhang, Mengdie Ding, Bo Xie, Yiming Tian, Xuan Wu, Lei Zuo, Xin Yang, Hongning Wang

**Affiliations:** 1 School of Life Science, Sichuan University, Animal Disease Prevention and Food Safety Key Laboratory of Sichuan Province, Key Laboratory of Bio-Resources and Eco-Environment, Ministry of Education, 29# Wangjiang Road, Chengdu 610064, China; 2014322040041@stu.scu.edu.cn (L.Z.); 2015322040022@stu.scu.edu.cn (Y.T.); 2016322040030@stu.scu.edu.cn (X.W.); 2017322040037@stu.scu.edu.cn (L.Z.); yangxin0822@163.com (X.Y.); 2 Sichuan Animal Science Academy, Sichuan Provincial Key laboratory of Animal Breeding and Genetics, Chengdu 610066, China; angelina_0708@hotmail.com; 3 Sichuan Provincial Center for Animal Disease Control and Prevention, Wuhou District, Chengdu 610041, China; zhangyidulin@163.com (Y.Z.); dingmengdie1987@126.com (M.D.); 4 Chengdu Chia Tai Agro-industry & Food Co., Ltd., Animal Healthy Disease Service, Gongping Town, Wenjiang District, Chengdu 610081, China; bobosky123@163.com

**Keywords:** porcine reproductive and respiratory syndrome virus, evolutionary, lineage, recombination

## Abstract

Recombination among porcine reproductive and respiratory syndrome viruses (PRRSVs) is thought to contribute to the emergence of new PRRSV variants. In this study, two newly emerged PRRSV strains, designated SCcd16 and SCya17, are isolated from lung tissues of piglets in Southwestern China. Genome comparative analysis reveals that SCcd16/SCya17 exhibit 93.1%/93.2%, 86.9%/87.0%, 85.3%/85.7%, and 83.6%/82.0% nucleotide similarity to PRRSVs JXA1, VR-2332, QYYZ and NADC30, respectively. They only exhibit 44.8%/45.1% sequence identity with LV (PRRSV-1), indicating that both emergent strains belong to the PRRSV-2 genotype. Genomic sequence alignment shows that SCcd16 and SCya17 have the same discontinuous 30-amino acid (aa) deletion in Nsp2 of the highly pathogenic Chinese PRRSV strain JXA1, when compared to strain VR-2332. Notably, SCya17 shows a unique 5-nt deletion in its 3’-UTR. Phylogenetic analysis shows that both of the isolates are classified in the QYYZ-like lineage based on ORF5 genotyping, whereas they appear to constitute an inter-lineage between JXA1-like and QYYZ-like lineages based on their genomic sequences. Furthermore, recombination analyses reveal that the two newly emerged PRRSV isolates share the same novel recombination pattern. They have both likely originated from multiple recombination events between lineage 8 (JXA1-like), lineage 1 (NADC30-like), and lineage 3 (QYYZ-like) strains that have circulated in China recently. The genomic data from SCcd16 and SCya17 indicate that there is on going evolution of PRRSV field strains through genetic recombination, leading to outbreaks in the pig populations in Southwestern China.

## 1. Introduction

Porcine reproductive and respiratory syndrome (PRRS) is a major viral disease, which is of significant economic importance affecting the global swine industry. The causative agent, PRRS virus (PRRSV), causes reproductive failure in pregnant sows and respiratory disease in pigs of all ages [1,2]. PRRSV is a member of the genus Porartevirus, Arteriviridae family, of the Nidovirales order [3,4]. The genome consists of a single molecule of single-stranded and positive-sense RNA with approximately 15 kilobases (kb) in length, which is transcribed into a set of 3′ co-terminal subgenomic mRNAs (mRNA1–mRNA7) responsible for the production of structural and non-structural proteins [3]. The genome of PRRSV encodes at least 10 open reading frames (ORFs), including ORF1a, 1b, 2a, 2b, 3, 4, 5a, and 5–7, flanked by two untranslated regions (UTRs), 5′-UTR and 3′-UTR. The virion contains eight structural proteins: five minor envelope proteins (GP2a, E, GP3, GP4 and GP5a), two major envelope proteins (GP5 and M) and the nucleocapsid protein (N) [5,6,7].

PRRSV is mainly divided into two genotypes: PRRSV-1 (a prototype strain Lelystad virus) and PRRSV-2 (a prototype strain VR-2332) [8]. Despite the fact that the two types of PRRSV cause similar clinical symptoms in infected pigs, they only exhibit about 50–60% nucleotide sequence identity [6]. In China, PRRSV-2 strains have been circulating and have been predominant in the field since their initial emergence in 1996 [9]. Based on the global PRRSV classification system and ORF5 sequence, PRRSV-2 was divided into nine lineages with several sublineages in each lineage [10,11]. To date, an overwhelming majority of the PRRSV-2 strains in China can be divided into four lineages: lineage 8/sublineage 8.7 (JXA1-like and CH-1a-like), lineage 5/sublineage 5.1 (VR-2332-like), lineage 3 (QYYZ-like) and lineage 1 (NADC30-like) [10,12]. Lineage 8 strains have been predominant since the initial emergence of PRRSV in China and include classical PRRSV (C-PRRSV) strains (CH-1a-like) prevalent before 2006 and highly pathogenic PRRSV (HP-PRRSV) strains (JXA1-like) prevalent after 2006 [9,13]. Although lineage 5 strains (VR-2332-like) appeared as early as 1996, they have not been pandemic in China [14]. Lineage 3 strains (QYYZ-like) are other variants that have emerged since 2010 and mainly circulate in Southern China [15]. Lineage 1 strains (NADC30-like) have been observed throughout China since 2013, and now their clinical detection rate is comparable to JXA1-like strains [16,17].

PRRSVs are prone to genetic evolution through the accumulation of mutations and recombinations among members of lineages/sublineages [13,18,19,20,21]. For example, lineage 1 (NADC30-like) PRRSV strains were reported to recombine with lineage 8 (JXA1-like) strains (HENAN-HEB, JL580, FJ1402 and HNhx) or lineage 5 (VR-2332-like) strains (Chsx1401, HENAN-XINX and HNyc15) [18,19,21,22,23,24]. Lineage 3 (QYYZ-like) PRRSV strains recombined with lineage 8 (JXA1-like) strains GM2, GD1404 and GDsg [15,25,26]. Recently, two PRRSV-2 strains, SCcd17 and SDhz1512, were reported to originate from recombination events among three lineages (Lineage 8, 5 and 1) [27,28]. In this study, porcine alveolar macrophage cells (PAMs) were used to isolate two novel recombinant PRRSV strains, SCcd16 and SCya17, from piglets in Sichuan province, Southwestern China, during 2016–2017. The two newly emerged PRRSV isolates have a very complex genetic background. They are likely products of multiple recombination events among lineage 8 (JXA1-like), lineage 1 (NADC30-like) and lineage 3 (QYYZ-like) strains that have circulated in Southwestern China recently, which differ from the previously reported recombination patterns from 2011 to 2017.

## 2. Materials and Methods

### 2.1. Sample Collection

Clinical samples, including lungs and lymph nodes, were collected from diseased piglets at two different pig farms in Sichuan province in Southwestern China during 2016–2017. The affected pigs had a high fever (40.2–42.1 °C) and exhibited overt signs of respiratory illness. Morbidity rates were 56.2%/45.3% (SCcd16/SCya17) and mortality rates were 7.3%/4.8%. To fully understand the evolutionary process of the two PRRS viruses that emerged in Southwestern China and to aid prevention and control policies against the disease, a genomic scale analysis was necessary. Clinical tissues were homogenized in an RPMI-1640 medium (Transgene, Beijing, China) using TissueLyser (Beijing, China) for RNA extraction and virus isolation. The samples that were not analyzed immediately were kept at −70 °C until use.

### 2.2. Virus Isolation

The tissue homogenates were centrifuged at 5000× *g* for 10 min. The suspensions were passed through 0.22-mm filters and inoculated into PAMs. The collection of PAMs and virus-containing tissue samples in this study was performed in strict accordance with the guidelines for the care and use of laboratory animals approved by the Animal Ethics Committee (AEC) of Sichuan University (Chengdu, China, 15 January 2017, SYXK [Chuan] 2013-185). The inoculated cells were cultured in the RPMI-1640 medium supplemented with 5% FBS (HyClone, South Logan, UT, USA) at 37 °C in a humidified 5% CO_2_ atmosphere and monitored daily for cytopathic effects (CPE). Indirect immunofluorescence assay (IFA) was performed by using specific PRRSV monoclonal antibody against the PRRSV N protein (GeneTex, Irvine, CA, USA) and goat anti-rabbit IgG antibody conjugated with FITC (Proteintech, Rosemont, IL, USA) as the primary and second antibodies, respectively. Viruses from both strains were purified by plaque assay as previously described [29], and then the purified viral isolates were used to sequence their whole genomes.

### 2.3. Viral Genome Extraction, RACE and RT-PCR

Total RNA was extracted from the suspensions of PRRSV infected cells using the Trizol reagent (Invitrogen, Carlsbad, CA, USA). Reverse transcription (RT) was performed with the PrimeScript^TM^ RT Reagent Kit (TaKaRa, Dalian, China). Fourteen pairs of primers for PRRSV-2 spanning the entire viral genome were used for amplifying the complete genomes of SCcd16 and SCya17, as previously described [29]. The PCR products were purified and inserted into a pMD19T-simple vector (TaKaRa, Dalian, China), and at least 3 positive clones of each fragment were sent to the Shanghai Sangon Biological Engineering Technology and Services Co. (Shanghai, China) to determine a consensus sequence (at least 3 times). A deletion was found in the 3’-UTR of SCya17 and confirmed by repeated sequencing (6 times). The 5′/3′ end of the viral genome of each isolate was amplified using a 5′/3′ RACE kit (TaKaRa, Dalian, China).

### 2.4. Genome Alignment and Phylogenetic Analysis

The sequences of PRRSV ORFs and deduced proteins were analyzed using the MegAlign program with DNAstar 7.0 software (DNASTAR Inc., Madison, WI, USA). The phylogenetic analysis was conducted using the MEGA 6 (v6.06) (www. megasoftware.net) with bootstrap values (1000 replicates), the Kimura 2-parameter and a nucleotide substitution model [30]. For the construction of the neighbor joining phylogenetic tree, sequences were aligned using the ClustalW multiple alignment algorithm (http://www.clustal.org/clustal2/). Forty reference genomic sequences were downloaded from GenBank (Supplementary Table S1).

### 2.5. Recombinant Analysis

The complete genomic sequences of the two new PRRSV field strains (i.e., SCcd16 and SCya17) and another four representative strains (i.e., JXA1, VR-2332, FJFS/XJzx1-2015 (QYYZ-like) and NADC30) were aligned using the ClustaW program of the MEGA 6, and analyzed using SimPlot (v3.5.1, Baltimore, MD, USA) with a 200-bp window sliding along the genome (20-bp step size). Furthermore, the Recombination Detection Program 4 (RDP4, v4.24) [31] that contains seven algorithms (RDP, Bootscan, GENECONV, MaxChi, Chimaera, SiScan and 3Seq) was used to confirm the putative recombination events and precise recombination breakpoints. Recombination events were only considered significant (*p*-value ≤ 1 × 10^−6^) when supported by at least five of the seven detection methods. These breakpoints were used to divide the genomic sequences into segments for phylogenetic tree construction.

## 3. Results

### 3.1. Virus Isolation

**Figure 1:**
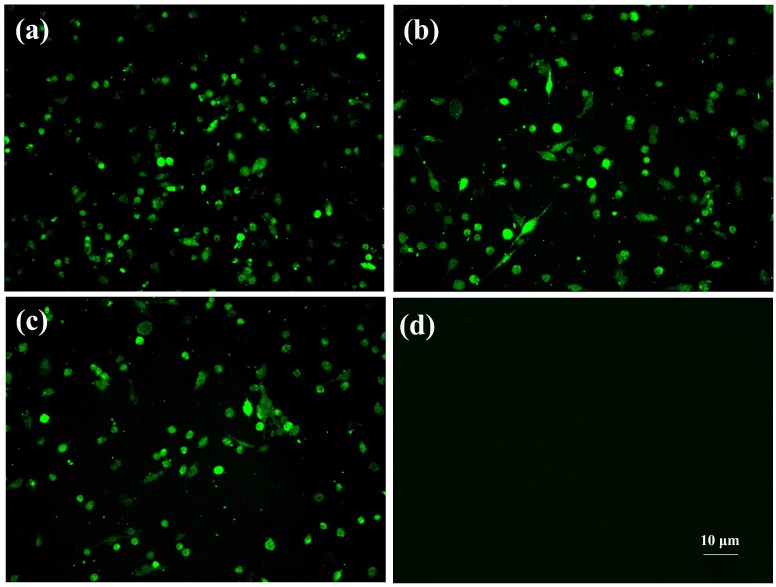
The results of the IFA. (**a**) PAMs inoculated with SCcd16; (**b**) PAMs inoculated with SCya17; (**c**) positive control cells inoculated with SCwhn09CD; (**d**) uninfected negative controls for PAMs. Scale bar = 10 μm.

The tissue suspensions that SCcd16 and SCya17 were isolated from were inoculated into PAMs, and the strain SCwhn09CD (isolated in 2009) was used as a positive control in this assay. The results from IFA showed fluorescence signals observed in all three cultures inoculated with PRRSV strains, SCcd16, SCya17, and SCwhn09CD, indicating that the two new strains, SCcd16 and SCya17, were successfully isolated from the clinical samples. No obvious CPEs were observed and the IFA results were negative in the control cells (Figure 1).

### 3.2. Genomic Characterization and Homology Analysis

The complete genomic sequences of SCcd16 and SCya17 were determined and deposited in the GenBank database under the accession numbers of MF196905 and MH324400, respectively. The genomes of SCcd16 and SCya17 were 15,321 and 15,315 nucleotides (nt) in length, respectively, excluding the poly(A) tail at the 3′ end. SCcd16/SCya17 shared 93.1%/93.2%, 86.9%/87.0%, 85.3%/85.7% and 83.6%/82.0% sequence identity with JXA1 (lineage 8), VR-2332 (lineage 5), QYYZ (lineage 3) and NADC30 (lineage 1), respectively (Table 1). Their sequence identity with LV (PRRSV-1) was only 44.8%/45.1%, indicating that both SCcd16 and SCya17 belong to the PRRSV-2 genotype.

In order to examine the genomic variation in the two novel PRRSV isolates, the nucleotide and amino acid (aa) homology of the 5′-UTR, 3′-UTR, ORF1a, ORF1b and ORF2–7 genes in the two isolates and in four representative strains of PRRSV-2 (JXA1, VR2332, QYYZ and NADC30) were compared. The results showed that ORF1a, ORF1b and the 3′-UTR of SCcd16 shared 93.4–99.5% nucleotide (95.3–97.7% aa) homology with JXA1, which was higher than the homology shared with VR-2332, QYYZ and NADC30. ORF2–4 of the SCcd16 isolate shared 90.5–94.2% nucleotide (87.2–93.4% aa) identity with NADC30, a higher percentage than that with the other strains, and ORF5–7 of the SCcd16 isolate shared 93.4–95.0% nucleotide (93.0–97.1% aa) identity with QYYZ, which was higher than that with the others. In addition, its 5′-UTR region shared 91.4% nucleotide homology with VR-2332, which was higher than that with the other three strains. The SCya17 strain’s 5′-UTR and ORF3–4 shared 88.0–88.7% nucleotide (88.2–90.5% aa) homology with VR-2332, which was higher than that with the other strains, whereas SCya17 ORF1a, ORF1b, and 3′-UTR shared 94.6–97.4% nucleotide (93.1–97.7% aa) identity with JXA1, which was higher than that with the others. SCya17 ORF5–7 shared 92.0–93.5% nucleotide (94.0–98.3% aa) identity with QYYZ. In addition, its ORF2 region shared 89.8% nucleotide homology (92.2% aa) with NADC30, which was higher than that with the other three strains (Table 1).

### 3.3. Phylogenetic Analysis

**Figure 2:**
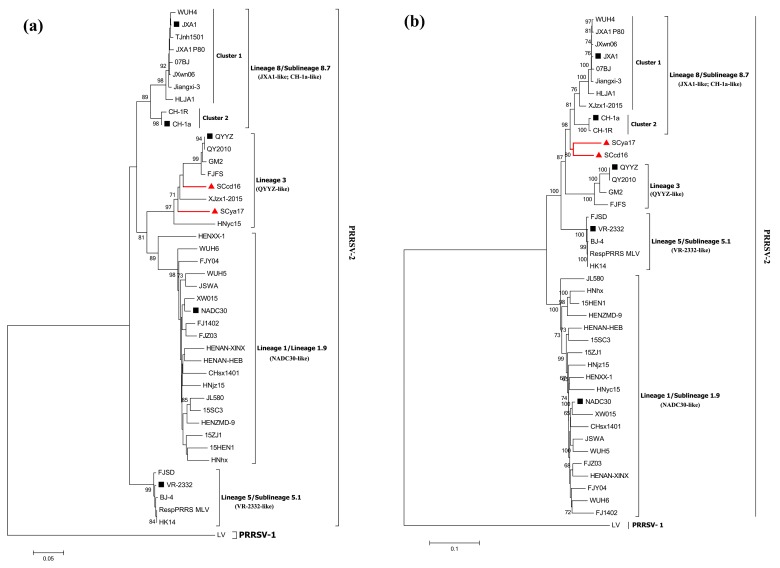
Phylogenetic trees based on ORF5 (**a**) and full-length genomic sequence (**b**) of SCcd16 and SCya17 isolates with 40 PRRSV reference strains available in GenBank. The two isolates in this study are labeled with “red triangle”. The representative strains are labeled with “black squares”. The phylogenetic tree is constructed by using the N-J method (1000 bootstrap) in MEGA6. Numbers along branches are bootstrap values. Scale bar indicates nucleotide substitute per site.

To establish the genetic relationships of SCcd16 and SCya17 with strains of PRRSVs, we constructed phylogenetic trees based on ORF5 and the complete genomic sequences of 40 reference PRRSVs available in GenBank (Supplementary Table S1). The results showed that all of the PRRSV-2 strains in China belonged to one of four lineages based on the ORF5 genotyping: lineage 8 (JXA1-like and CH-1a-like), lineage 5 (VR-2332-like), lineage 3 (QYYZ-like) and lineage 1 (NADC30-like) (Figure 2a). All of the lineage 8 (8.7) PRRSV strains reported in China since 1996 showed evidence of evolutionary divergence, and therefore, have been further classified into two clusters (clusters 1 and 2). Cluster 1 consists of HP-PRRSV-like strains, while cluster 2 consists of Chinese C-PRRSV strains. Notably, the SCcd16 and SCya17 isolates were classified in lineage 3 based on ORF5 genotyping, whereas they belonged to an inter-lineage based on their whole genomes. This inter-lineage was located between lineage 8, represented by JXA1 and CH-1a, and lineage 3, represented by QYYZ (Figure 2b). These results may indicate that mosaic recombination events occurred in the genomes of the two isolates.

### 3.4. Sequence Analysis of Nsp2, GP5 and 3′-UTR

**Figure 3:**
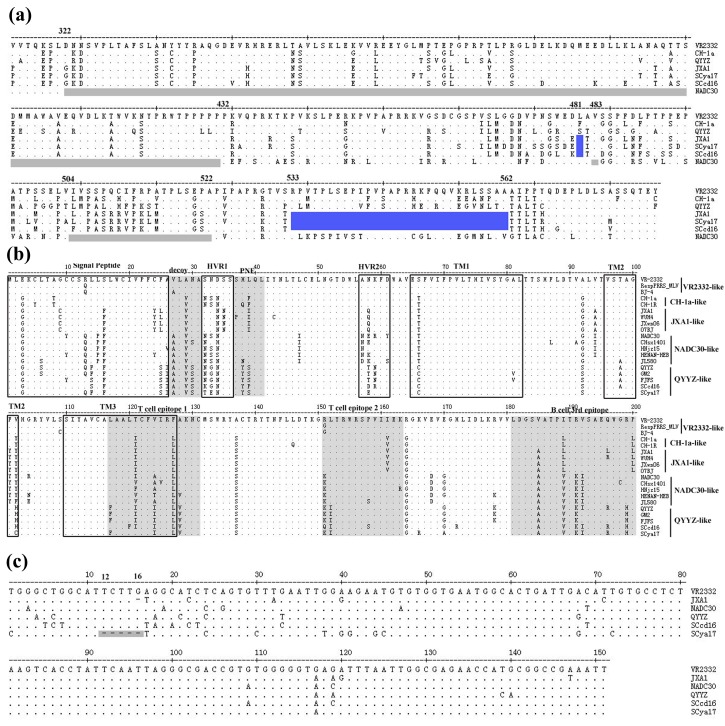
The sequence alignment of Nsp2, GP5, and 3′-UTR. (**a**) Two discontinuous amino acid deletions (481 and 533–562) in Nsp2 of SCcd16 and SCya17 (blue regions). Three discontinuous amino acid deletions (322–432, 483, and 504–522) in Nsp2 of NADC30 (gray regions); (**b**) multiple alignment of GP5 amino acid sequences of SCcd16 and SCya17 and seventeen PRRSV reference strains. Black boxes indicate the regions of signal peptide, two hypervariable regions (HVRs), and three transmembrane domains (TMs). Gray areas indicate the amino acid residues in the decoy epitope, primary neutralizing epitope (PNE), two T cell epitopes and the 3rd B cell epitope; (**c**) five continuous nucleotides deletion in 3′-UTR at positions 12 to 16 of SCya17 (gray region).

**Figure 4:**
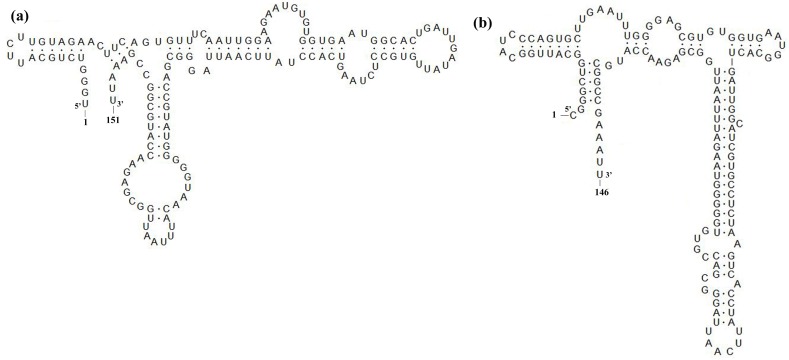
The predicted secondary structures of 3′-UTR sequences of SCcd16 (**a**) and SCya17 (**b**) are presented. The positions of the first and last nucleotide of both the 5′ and 3′-UTR are indicated. Schematic representation of secondary structures predicted by Mfold (http://mfold.rit.albany.edu/?q=mfold/RNA-Folding-Form) under default folding conditions and modified using RNAviz (http://rnaviz.sourceforge. net/).

Amino acid alignment of the nonstructural protein 2 (Nsp2) of SCcd16 and SCya17 isolates with the representative strains showed that the two strains had the same amino acid deletions as JXA1 (an HP-PRRSV strain identified in China in 2006). These deletions were identified as a discontinuous 30-aa deletion (1-aa at position 481, and 29-aa at positions 533 to 562) when compared with the sequence of VR-2332 (Figure 3a). In addition, comparisons of the amino acid analyses of SCcd16 and SCya17 GP5 with those of the other representative strains showed that both isolates were more closely related to QYYZ-like strains than to the other strains. Several unique amino acids, such as F/S^14^, S^25^, I^26^, Y^38^, S/P^38^, S^92^, A^98^, H/C^102^, F^117^, I^124^, I^152^ and H^199^, were only identified in QYYZ-like strains, including SCcd16 and SCya17 (Figure 3b). Moreover, a majority of the amino acid substitutions lied within the signal peptide and hypervariable regions (HVRs) domain of the protein (Figure 3c). Interestingly, SCya17 exhibited a novel continuous 5-nt deletion in its 3′-UTR at positions 12 to 16, which has not been reported previously. In comparison with that of an intact 3′-UTR (SCcd16), SCya17 is predicted to have an altered secondary structure in its 3′-UTR due to its unique 5-nt deletion (Figure 4).

### 3.5. Recombination Analysis

**Figure 5:**
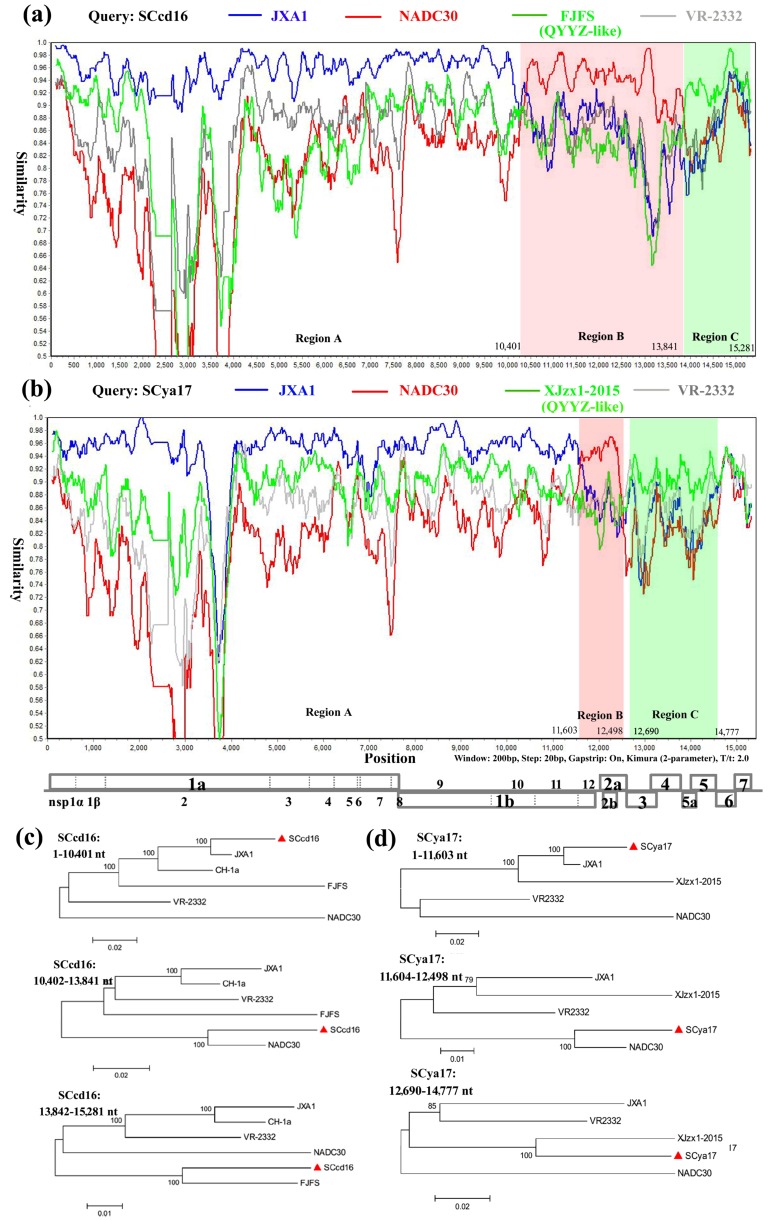
Recombination analysis of strain SCcd16 and SCya17. Analysis is made use of a sliding window of 200-bp window and a 20-bp step. The *y*-axis indicates the percentage similarity between the query sequence and the reference sequences. (**a**) Genome scale similarity comparisons of SCcd16 (query) with JXA1 (blue), NADC30 (red), FJFS (QYYZ-like, green) and VR-2332 (gray); (**b**) genome scale similarity comparisons of SCya17 (query) with JXA1 (blue), NADC30 (red), XJzx1-2015 (QYYZ-like, green) and VR-2332 (gray). The supposed recombination regions are shown with different colors, and the recombination breakpoints are marked at the bottom with nucleotide sites and viral genome structure referenced to VR-2332; (**c**) phylogenetic trees based on different regions of SCcd16; (**d**) phylogenetic trees based on different regions of SCya17.

To identify possible recombination events, we detected recombination using SimPlot and RDP4 software. The analysis revealed that SCcd16 and SCya17 sequences showed remarkably high degrees of certainty, with *p*-values of ≤ 1 × 10^−6^, from the results of at least five detection methods (Table 2). From the similarity plot, three recombination breakpoints within the SCcd16 genome were identified, which were located in nsp10 (nt 10,401), ORF5 (nt 13,841), and ORF7 (nt 15,281) (Figure 5a). The breakpoints in SCcd16 separated its genome into three regions, where region A (nt 1–10,401) was closely related to the JXA1-like strain, region B (nt 10,402–13,841) was closely related to NADC30-like strain, and region C (nt 13,842–15,281) was closely related to the FJFS (QYYZ-like) strain (Figure 5c). Four recombination breakpoints were identified in SCya17, which were located in nsp12 (nt 11,603), ORF2 (nt 12,498), ORF3 (nt 12,690), and ORF6 (nt 14,777) (Figure 5b). The breakpoints in SCya17 separated its genome into three main regions, where region A (nt 1–11,603) was closely related to the JXA1-like strain, region B (nt 11,604–12,498) was closely related to NADC30-like strain, and region C (nt 12,727–14,582) was closely related to the XJzx1-2015 (QYYZ-like) strain (Figure 5d). These results indicated that the SCcd16 and SCya17 strains shared the same recombination pattern. They have both likely originated from multiple recombination events among lineage 8 (JXA1-like), lineage 1 (NADC30-like) and lineage 3 (QYYZ-like) strains.

## 4. Discussion

HP-PRRSV continues to be a serious threat to the Chinese swine industry since its initial outbreak in large areas of China in 2006 [13,32]. Numerous studies indicate that a majority of the HP-PRRSV (JXA1-like) strains isolated in China during 2007–2012 were seeded by that outbreak [32,33,34,35]. However, with the emergence of new lineage 3 (QYYZ-like) viruses in 2010 and lineage 1 (NADC30-like) viruses in 2013, PRRSV strains in China were reported to show different patterns of recombination between members of lineages/sublineages [15,18,19,24,25,26,27]. According to this study, two new PRRSV isolates, SCcd16 and SCya17, belong to lineage 3 strains based on global genotyping. Strikingly, recombination analysis based on their whole genome sequences revealed that the two isolates share the same novel recombination pattern, they both have originated from multiple recombination events among lineage 8 (JXA1-like), lineage 1 (NADC30-like) and lineage 3 (QYYZ-like) strains that have circulated in China recently. This new recombination pattern of the two isolates (SCcd16 and SCya17) is different from the previously identified PRRSV strains isolated in China from 2011 to 2017 (Table 3), indicating that emergence of new PRRSV recombination variants in China has taken place.

Recombination and mutation are the principal mechanisms of PRRSV evolution, and the two types of events have further increased the genetic diversity and complexity of PRRSVs in China [10,36,37]. Phylogenetic analysis based on global genotyping indicated that PRRSV-2 strains in China were mainly clustered into four lineages: lineage 8 (sublineage 8.7), 5 (5.1), 3 and 1 (1.9). Furthermore, lineage 8 (8.7) strains in China could be further classified into two clusters: JXA1-like and CH-1a-like strains. Lineage 1 strains (NADC30-like) have undergone rapid genomic evolutionary divergence, since they first emerged in China in 2013, due to a high incidence of recombination [12,19,38]. They can be further divided into two branches: the main branch consists of the majority of NADC30-like strains in China, and the minor branch consists of the other three strains (HNhx, 15HEN1, and HENZMD-9) (Figure 2b). The uptake of nucleotide sequences from JXA1-like, NADC30-like and QYYZ-like strains by the genomes of SCcd16 and SCya17 leads to the constitution of an inter-lineage between lineage 8 and lineage 3, which they belong to, based on their genomic sequences. These results indicated the presence of PRRSV strains with genetic diversity and complexity in China.

The 3′-UTR of PRRSV plays important roles in replication, transcription and infectivity [39]. Sequence alignment of the 3′-UTR of SCya17 with other strains showed that SCya17 has a novel continuous 5-nt deletion in its 3′-UTR at positions 12 to 16 (Figure 1c), which is reported here for the first time. Previous studies indicated that the sequences of 5′-UTR and 3′-UTR are involved in viral virulence [40,41,42]. Whether the deletion in 3′-UTR of SCya17 is associated with its virulence requires further study. Sun et al. demonstrated that the 40 nucleotides following the ORF7 stop codon are dispensable for the viability of the PRRSV-2 virus [40]. Therefore, the 5-nt deletion in the SCya17 resulted from a natural selection event that did not affect the viability of the newly emerged PRRSV variant.

The presence of the QYYZ-like PRRSV strains (QYYZ and GM2) was first reported in Southern Mainland China. Pathogenicity analysis revealed that the QYYZ-like PRRSVs were of low pathogenicity with mild clinical presentations [15]. NADC30 was initially identified in the United States in 2008, and also belongs to a moderately virulent strain, as evaluated by in vivo experiments [43]. Subsequently, the strain was introduced into China through the transportation of pigs or pig products from North America to China [18], and the Chinese NADC30-like (HENAN-HEB, HENAN-XINX, and CHsx1401) strains were initially identified during 2013–2014 [16]. Previous studies have revealed that increased PRRSV virulence is closely related to the recombination among different strains. For example, the QYYZ-like PRRSV strain, GDsg that recombined with the HP-PRRSV-derived commercial vaccine (JXA1-P80) was reported to be more virulent in pigs than its parental strain QYYZ [44]. Two NADC30-like PRRSV strains, JL580 and FJ1402, which recombined with HP-PRRSV-like strains (09HEN1/GD), were demonstrated to be highly pathogenic strains in experiments in animals [18,23]. The two newly emerged strains, SCcd16 and SCya17, reported in this study originated from recombination among three lineages. Considering the low mortality (4.8–7.3%) caused by the two PRRSV isolates, they might belong to PRRSV strains of low pathogenicity, however, the virulence of the two strains needs to be further evaluated in experiments with pigs.

## 5. Conclusions

In summary, two PRRSV strains, SCcd16 and SCya17, were isolated from lung tissues of piglets in Southwestern China. Recombination analysis revealed that the two newly emerged PRRSV isolates shared the same novel recombination pattern. They both likely resulted from multiple recombination events among lineage 8 (JXA1-like), lineage 1 (NADC30-like) and lineage 3 (QYYZ-like) strains that have circulated in China recently. Our study highlights the importance of continuous monitoring of PRRSVs in China and the necessity for new vaccine development.

## Figures and Tables

**Table 1:** Genome positions, protein sizes, and nucleotide and amino acid identities of different regions of SCcd16 and SCya17 compared with other PRRSV-2 representative strains.

Region	Length (nt/aa)						Pairwise % Identity (nt/aa)			
	JXA1	VR-2332	QYYZ	NADC30	SCcd16	SCya17	JXA1 ^1^	VR2332 ^1^	QYYZ^1^	NADC30 ^1^
Complete genome	15,319	15,451	15,526	15,047	15,321	15,315	93.1 *	86.9 *	85.3 *	83.6 *
							93.2 **	87.0 **	85.7 **	82.0 **
5′UTR	189	189	190	191	189	188	89.4	**91.4**	89.4	90.7
							87.4	**88.7**	85.4	85.4
ORF1a	7422/2475	7512/2502	7614/2538	7119/2375	7422/2475	7422/2475	**96.0/95.3**	85.3/84.2	81.8/82.7	76.7/79.1
							**94.6/93.1**	84.8/83.7	81.8/83.1	76.6/79.0
ORF1b	4383/1462	4383/1462	4383/1462	4383/1462	4383/1462	4383/1462	**93.4/97.7**	89.3/95.8	88.8/95.9	90.1/97.1
							**95.3/97.7**	89.3/95.9	89.0/95.6	87.7/95.9
ORF2	771/256	771/256	771/256	771/256	771/256	771/256	86.5/85.6	87.7/88.7	87.4/89.9	**94.2/93.4**
							86.9/86.0	89.4/88.7	89.4/90.7	**89.8/92.2**
ORF3	765/254	765/254	765/254	765/254	765/254	765/254	80.9/74.9	81.8/76.9	79.2/75.3	**92.9/87.5**
							85.5/83.5	**88.0/88.2**	87.2/85.1	83.5/81.6
ORF4	537/178	537/178	537/178	537/178	537/178	537/178	82.7/77.1	85.7/78.2	83.6/78.2	**90.5/87.2**
							86.8/88.8	**88.6/90.5**	87.7/89.9	85.3/86.6
ORF5	603/200	603/200	603/200	603/200	603/200	603/200	84.2/82.1	84.6/81.1	**93.4/93.0**	85.4/83.6
							83.7/82.6	85.1/82.6	**92.0/94.0**	83.6/84.6
ORF6	525/174	525/174	525/174	525/174	525/174	525/174	91.2/94.3	92.2/95.4	**95.0/97.1**	89.1/92.0
							91.4/95.4	92.0/95.4	**93.5/98.3**	89.7/93.1
ORF7	372/123	372/123	372/123	372/123	372/123	372/123	90.9/89.5	93.0/92.7	**93.5/95.2**	89.2/89.5
							89.8/89.5	91.7/91.1	**92.2/94.4**	88.7/90.3
3′UTR	150	151	151	151	151	146	**99.5**	92.1	94.2	92.1
							**97.4**	91.6	93.7	89.5

^1^ In each box, the upper figure is the percentage identity with SCcd16 (*) and the lower figure is the percentage identity with SCya17 (**). The highest nucleotide and amino acid identities of different regions are indicated in bold typeface.

**Table 2:** Information on recombination events detected in SCcd16 and SCya17.

Recombinant Strain	Breakpoints		Parental Sequence		Detection Methods (*p*-Value)						
	Beginning	Ending	Minor	Major	RDP	GENECONV	BootScan	MaxChi	Chimaera	SiScan	3Seq
SCcd16	10,401	13,841	NADC30	JXA1	2.120 × 10^−51^	1.358 × 10^−13^	2.281 × 10^−52^	5.308 × 10^−21^	2.234 × 10^−23^	1.473 × 10^−32^	1.069 × 10^−120^
	13,842	15,281	FJFS	JXA1	5.691 × 10^−26^	NS	5.511 × 10^−16^	5.874 × 10^−19^	2.155 × 10^−11^	3.554 × 10^−21^	2.593 × 10^−34^
SCya17	11,603	12,498	NADC30	JXA1	2.845 × 10^−40^	3.323 × 10^−14^	5.417 × 10^−42^	7.336 × 10^−14^	1.030 × 10^−15^	1.684 × 10^−08^	1.907 × 10^−15^
	12,690	14,777	XJzx1-2015	JXA1	1.374 × 10^−49^	NS	5.013 × 10^−32^	2.658 × 10^−18^	2.064 × 10^−21^	8.414 × 10^−17^	3.349 × 10^−29^

NS: not significant.

**Table 3:** Recombination analysis of PRRSVs isolated in China during 2011–2017.

Strains	Isolation Date	Recombination Pattern (Major Parent + Minor Parent)	Virulence	Accession No.
GM2	2011	JXA1-P80 (lineage 8) + QYYZ (lineage 3)	Low Pathogenic	JN662424
HENAN-HEB	2013	NADC30 (lineage 1) + JXA1 (lineage 8)	-	KJ143621
HENAN-XINX	2013	NADC30 (lineage 1) + VR-2332 (lineage 5)	-	KF611905
CHsx1401	2014	NADC30 (lineage 1) + VR-2332 (lineage 5)	Moderately Pathogenic	KP861625
JL580	2014	NADC30 (lineage 1) + 09NEN1 (lineage 8)	Highly Pathogenic	KR706343
FJ1402	2014	NADC30 (lineage 1) + GD (lineage 8)	Highly Pathogenic	KX169191
GD1404	2014	JXA1-P80 (lineage 8) + QYYZ (lineage 3)	Highly Pathogenic	MF124329
GDsg	2015	JXA1-P80 (lineage 8) + QYYZ (lineage 3)	Moderately Pathogenic	KX621003
HNyc15	2015	NADC30 (lineage 1) + VR-2332 (lineage 5)	-	KT945018
15HEN1	2015	NADC30 (lineage 1) + JXA1-P80 (lineage 8)	-	KX815413
SDhz1512	2015	JXA1 (lineage 8) + VR-2332 (lineage 5) + NADC30 (lineage 1)	Low Pathogenic	KX980392
TJnh1501	2015	CHsx1401 (lineage 1) + TJbd14-1 (lineage 8)	Moderately Pathogenic	KX510269
HNhx	2016	NADC30 (lineage 1) + JXA1 (lineage 8)	-	KX766379
SCcd17	2017	NADC30 (lineage 1) + JXA1 (lineage 8) + VR-2332 (lineage 5)	-	MG914067
**SCcd16**	2016	JXA1 (lineage 8) + NADC30 (lineage 1) + QYYZ (lineage 3)	-	MF196905
**SCya17**	2017	JXA1 (lineage 8) + NADC30 (lineage 1) + QYYZ (lineage 3)	-	MH324400

“-” indicates the lack of experiment in vivo.

## Supplementary Materials

The following are available online at http://www.mdpi.com/1999-4915/10/6/328/s1, Table S1: Information on the 40 reference PRRSVs downloaded from GenBank.
