# Supplementary material for: Whole Genome Analysis of Two Novel Type 2 Porcine Reproductive and Respiratory Syndrome Viruses with Complex Genome Recombination between Lineage 8, 3, and 1 Strains Identified in Southwestern China
Source: Viruses. 2018 Jun 15;10(6):328. doi: 10.3390/v10060328 (PMC6024730; doi:10.3390/v10060328)
Supplement: Supplementary file 1 [file viruses-10-00328-s001.pdf]

Supplementary Table S1. Information on the 40 reference PRRSVs downloaded from GenBank.

Reference strains	Country/year	Accession No.	Reference strains	Country/year	Accession No.
Lelystad virus	EU/1991	M96262	HENAN-XINX	CHN/2013	KF611905
VR-2332	USA/1995	AY150564	JL580	CHN/2013	KR706343
CH-1a	CHN/1996	AY032626	XW015	USA/2013	KF724409
BJ-4	CHN/2000	AF331831	HLJA1	CHN/2013	KT351739
HK14	CHN/2004	KF287141	FJ1402	CHN/2014	KX169191
RespPRRS MLV	USA/2005	AF066183	JSWA	CHN/2014	KY373214
JXA1	CHN/2006	EF112445	HENXX-1	CHN/2014	KU950372
jiangxi-3	CHN/2007	EU200961	CHsx1401	CHN/2014	KP861625
07BJ	CHN/2007	FJ393459	XJzx1-2015	CHN/2015	KX689233
CH-1R	CHN/2008	EU807840	HNjz15	CHN/2015	KT945017
NADC30	USA/2008	JN654459	FJZ03	CHN/2015	KP860909
JXwn06	CHN/2008	EF641008	HNyc15	CHN/2015	KT945018
JXA1 P80	CHN/2008	FJ548853	FJY04	CHN/2015	KP860910
QY2010	CHN/2010	JQ743666	WUH5	CHN/2015	KU523366
QYYZ	CHN/2010	JQ308798	HENZMD-9	CHN/2015	KU950374
GM2	CHN/2011	JN662424	TJnh1501	CHN/2015	KX510269
WUH6	CHN/2011	KU523367	15ZJ1	CHN/2015	KX815432
FJFS	CHN/2012	KP998476	15SC3	CHN/2015	KX815428
HENAN-HEB	CHN/2012	KJ143621	FJSD	CHN/2015	KP998474
WUH4	CHN/2012	JQ326271	HNhx	CHN/2016	KX766379
